# Atypical neuroretinitis after exposure to tattoo ink

**Published:** 2019

**Authors:** Oana Roxana Scripcă, Coralia Calancea

**Affiliations:** * Calmedo, Brașov, Romania; ** Kronstadt optik, Brașov, Romania

**Keywords:** uveitis, pigment nanoparticles, ink tattoo, synchrotron X-ray fluorescence (XRF), lymphatic system aggression

## Abstract

**Purpose:** The paper aimed to highlight the ocular complications after exposure to tattoo ink by presenting a case report.

**Material and methods:** A 19-year-old patient presented to the ophthalmologist for decreased vision and photophobia after a tattoo she performed on her left thigh.

Tattoo ink is mentioned as an inflammatory process trigger in current literature at skin level, but the eye related complications are those that can cause the patient’s quality of life to plummet through important VA alteration.

Ocular inflammatory processes after exposure to tattoo ink can cause uveitis, patients may present with changes characteristic of Anterior Uveitis or may have significant ocular complications such as papillary swelling, retinal haemorrhage, and retinal macular effusion.

The presented case showed how difficult it is to name Neuroretinitis’ etiology in a situation in which laboratory and imaging investigations excluded most of the causes that could determine such an aggressive pathology in a young person.

Delayed hypersensitivity reaction caused by tattoo pigments is one of the mechanisms cited in the existing literature, but the mechanism that delineates ocular complications is very complex and, at this point, unfortunately little known.

## Introduction

The tattoo is practiced all around the world and dates back 5 300 years ago, with the oldest recorded human tattoo being found on a well-preserved mummy discovered in the Ötztal Alps in Italy [**1**]. Tattooing is an increasingly popular practice, regardless of age, gender, or social class, being an act of expressing unfulfilled emotional needs according to psychologists.

Although today little is known about the biochemical reactivity of ink particles with skin cells and tissues, what is definitely known is that tattoo ink goes more than skin deep, causing serious health problems.

## Case report

Attention is drawn to the case of a young woman (19 years old) who presented to the ophthalmological consultation claiming decreased vision and moderate photophobia. Her initial visual acuity (VA) was 20/ 25 not correctable and the Intraocular Pressure was 14 mmHg in RE (right eye) and 18 mmHg in LE (left eye).

The slit lamp examination of the anterior segment revealed bilateral discrete conjunctival congestion, fine keratic precipitates (KP) on LE in the inferior third of the cornea and two posterior synechia and some KP on her RE, no Tyndall effect was observed.

The fundus slit lamp examination at the time of the first presentation was without inflammatory changes on the vitreous, and her RE fundus examination was within normal limits. Fundus examination of the left eye revealed an optic disc with blurred margins; the retinal veins were dilated and tortuous, with no macular lesions or lesions in the peripheral retina.

She received treatment with mydriatic, systemic and topic corticotherapy and was recommended further laboratory investigations.

A few days after the first presentation, she returned accusing decreased vision. VA on LE was 20/ 35 and the VA for the RE 20/ 25. Examination of the posterior pole revealed bilateral papillary edema with flame-shaped hemorrhages and macular edema. At this point, it was decided to increase the dose of systemic corticosteroid.

She performed common blood tests such as CBC (complete blood count), erythrocyte sedimentation rate and inflammatory markers, all of which were normal. She was also recommended tests for rapid plasma reagin (RPR), TPHA, VDRL, rheumatoid factor, antinuclear antibodies, anti-double stranded DNA, C3, C4 complement and immune complexes circulantes (CIC), total protein test and serum protein electrophoresis, angiotensin-converting enzyme and once again they all were within normal limits. Serological tests were performed next in order to look for certain antibodies for CMV, Toxoplasma gondii, Toxocara canis, Herpes simplex, Epstein Barr, Rubella infection. They were also within limits.

On detailed questioning, she mentioned a tick bite ten years before without prophylactic antibiotic therapy. The screening for Lyme disease, Borrelia Burgdorferi Western Blot Ig M, and Ig G was negative. 

Because her mother died of complications of tuberculosis, Quantiferon TB gold test was performed and the results were negative.

To exclude Behçet’s Disease and ankylosing spondylitis, she was recommended HLA B51 and HLA B27, whose results were also negative. Radiological imaging investigation, such as Chest X-ray and spinal-sacroiliac joints, were also requested but they were normal.

At this point, the most commonly encountered factors that could cause neuroretinitis in a young person were excluded by laboratory and paraclinical investigations.

During the treatment with 32 mg methylprednisolone dose, the visual status of the patient improved and the aspect of the optic nerve papilla improved with the remission of papillary haemorrhages and retinal edema. A gradual corticosteroid dose decrease was initiated but below 12 mg, the papillary flame haemorrhage recurred, so that the maintenance dose remained 16 mg/ day.

**Fig. 1:**
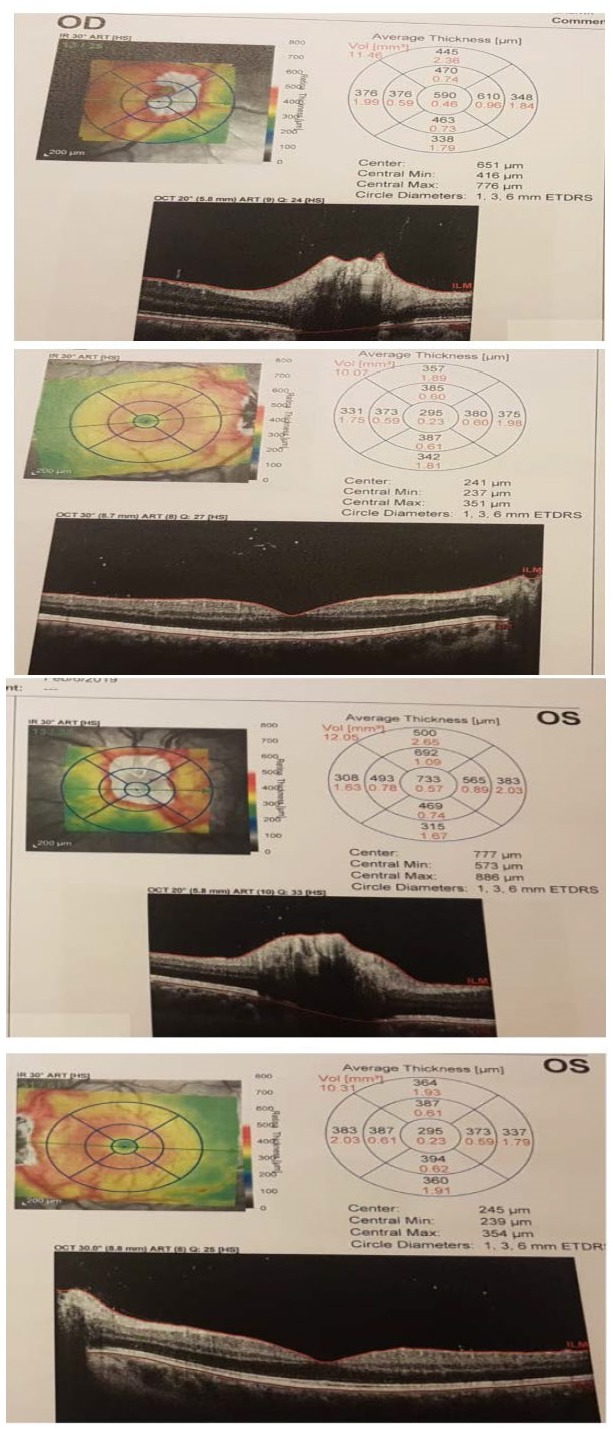
OCT for RE and LE revealing papillary edema without affecting Ganglion cell layer and secondary macular edema

OCT was performed revealing papillary edema without affecting Ganglion cell layer and secondary macular edema (**Fig. 1**).

Due to vision degradation and poor response to corticotherapy, the patient was guided to a multidisciplinary assessment.

Following the assessment by the infectious physician, further tests have been performed, namely HIV, Bartonella henselae, Chlamydia trachomatis, Hepatitis B and C. They were all negative.

The immunological screening profile excluded an autoimmune disease as the main element in the patient’s condition and the immunologist recommended the evaluation of metabolic functions during corticotherapy to avoid the occurrence of side effects caused by the treatment. 

Dermatoscopy of the black tattoo from the thigh showed the presence of several types of tattoo pigments without the structural modification of the epidermis.

Neurological evaluation was within normal limits without revealing neurological signs; brain contrast MRI was performed and a small arterio-venous malformation - fractionometric projected at subcortical cerebral cortex was observed, silent bleeding traces were detected without being associated with the status of the patient.

**Fig. 2:**
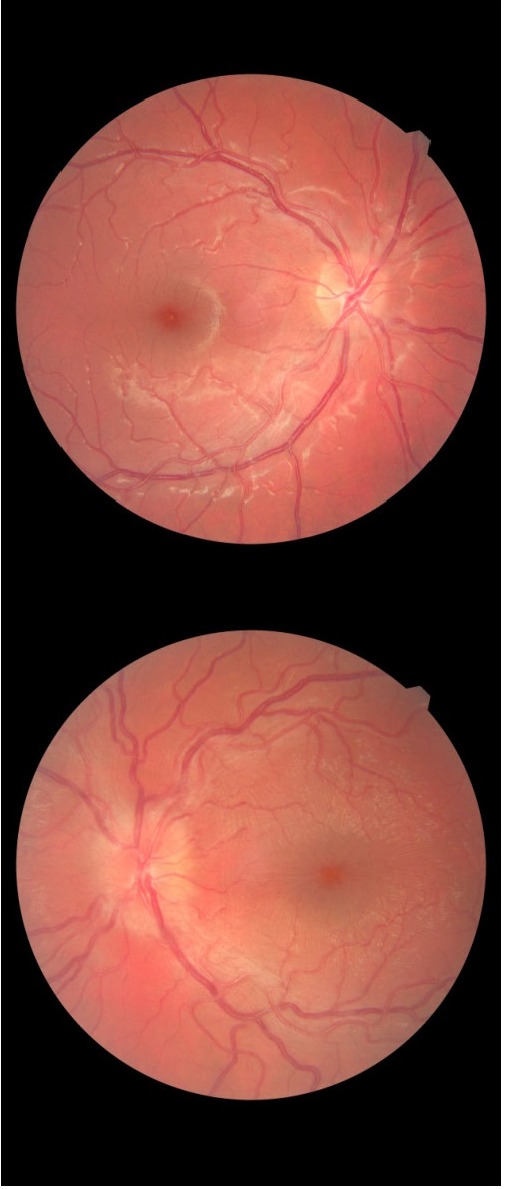
The fundus aspect for both eyes

**Fig. 3:**
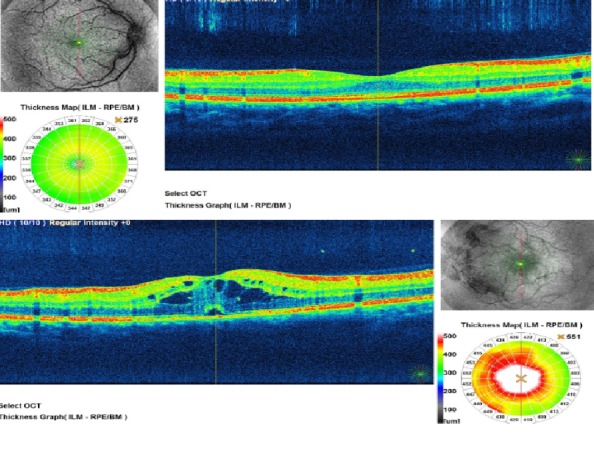
OCT aspect for both eyes. Multiple cyst-like (cystoid) areas of fluid in the macula were noticed in the left eye and caused retinal swelling

Every attempt to reduce corticotherapy below 12 mg/ day resulted in visual alteration and increased retinal and papillary edema. Unfortunately, after 3 months of retinotrophic and corticosteroid therapy, the sight has worsened (VA RE 16/ 20 and VA LE 8/ 20 not correctable) and the retina aspect as well (**Fig. 2**,**3**).

**Fig. 4:**
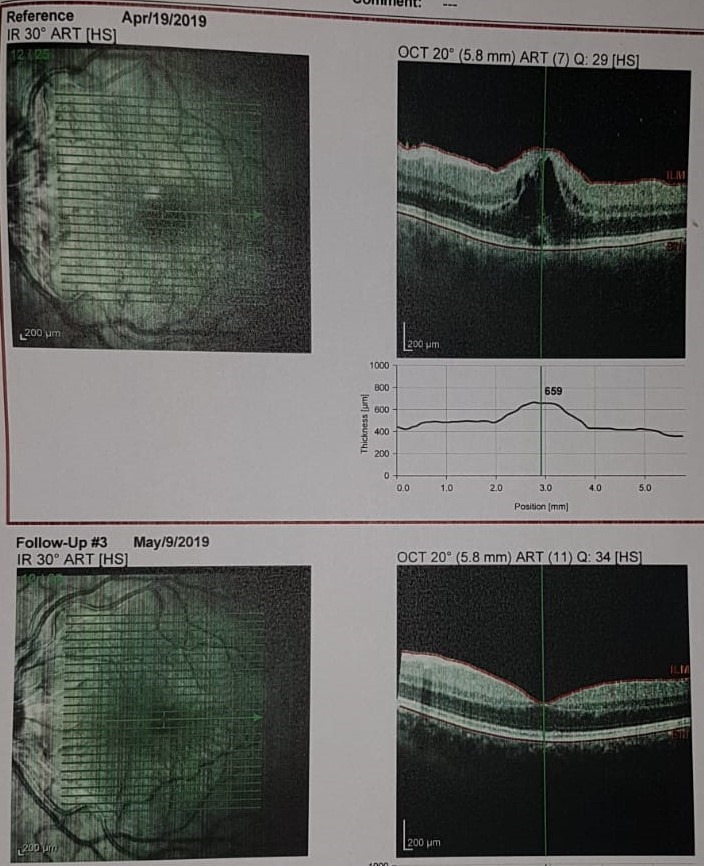
The OCT aspect in evolution for the Left Eye showing the remission of macular edema after intravitreal Triamcinolone

Due to cystoid macular edema and serous leakage to neuroepithelium, the patient received intravitreal triamcinolone under which the evolution was favorable with a reduction of retinal edema (**Fig. 4**).

Following the multidisciplinary assessment and analysis, it was concluded that the neuroretinitis is caused by the tattoo pigment.

The patient was asked which pigment was used for the tattooing and the search revealed that the ink had recall safety alert since 2014 because of the high amount of PAHs (polycyclic aromatic hydrocarbons) including naphthalene (measured values: naphthalene: 2.25 mg/ kg; total of PAHs: 2.38 mg/ kg). The Council of Europe Resolution ResAP (2008) on requirements and criteria for the safety of tattoos and permanent make-up recommends that the total amount of PAHs does not exceed 0.5 mg/ kg [**2**].

Currently, the sight of the patient is 18/ 20 both eyes, the fundus of her right eye is normal and a macular scar persists on the left eye, she is still on retinotrophic treatment, but it is without corticotherapy and she has not submitted any recurrences.

## Discussions

Neuroretinitis is a disease characterized by optic disc edema and macular subsequent edema. In 1977, Don Gass [**3**] found it to be due to increased permeability of disc vasculature with exudation of fluid into the peripapillary retinal.

Many of the etiologies are treatable conditions such as viral, bacterial, parasitic, or fungal neuroretinitis for which there is clear therapeutic management from the moment the infectious agent is identified by specific tests.

Autoimmune conditions can cause neuroretinitis but those can be excluded by performing laboratory testing such as antinuclear antibody test, angiotensin-converting enzyme, anti-double-stranded DNA, and C3 or human leukocyte antigen (HLA) typing [**4**].

Despite the wide variety of infectious and noninfectious disorders that may cause neuroretinitis, almost half of the cases evaluated by the ophthalmologist remain idiopathic.

An accurate diagnosis can result from a complete examination and a detailed anamnesis. Exposure history should be thoroughly explored, especially in young patients presenting atypical neuroretinitis, detailing aspects like medication use, recent travel, post vaccination status, unpasteurized and uncooked foods, sexual experience, animal contacts, and tattoos.

Existing literature cites anterior uveitis as one of the most frequent complications in tattooed patients; they tend to show a localized cutaneous response and the overall aspect of the tattoo changes, becoming raised and indurated simultaneous with ocular inflammation. In April 2018, the paper entitled “Tattoo associated retinochoroiditis” was published, in which the authors present the case of a young woman who was diagnosed with retinochoroiditis after tattooing [**5**].

The histological examination of the tattooed skin may reveal noncaseating granulomas suggesting an association with sarcoidosis or a reaction of a foreign body due to metallic elements or other elements of the injected substance (delayed hypersensitivity reaction) [**6**,**7**].

During tattooing, the ink is transported via an electric vibrating device from the skin surface into the derma where the pigment is deposited. For a long time, it was considered that the ink remains in the dermis without migrating to other structures but the researchers have demonstrated that tattoo ink travels to other parts of the body, especially the lymph nodes [**8**].

The study “Synchrotron-based ν-XRF mapping and μ-FTIR microscopy enable to look into the fate and effects of tattoo pigments in human skin” [**9**], published in 2017, describes in fine detail aspects of the biokinetics and chemical composition of tattoo ink. The content of nanoparticles in tattoo ink is little studied and unfortunately, there is no concrete data on what structures are affected by their migration in the body, so aspects of nanotoxicology are still being investigated by the researchers [**10**,**11**].

The paper highlights aspects related to the etiology of a neuroretinitis post tattooing in a young woman. Although existing literature contains signals that draw attention to the association of tattoo ink and the appearance of uveitis episodes, more studies are needed in order to detail the pathophysiological aspects and the mechanisms through which the blood-retina barrier integrity is affected by the nanoparticles present in tattoo ink. This will lead to a better management of atypical neuroretinitis associated with exposure to this type of dye.
